# Identification of prostaglandin receptors in human ureters

**DOI:** 10.1186/1471-2490-12-35

**Published:** 2012-12-10

**Authors:** Matthias Oll, Claudia Baumann, Turang E Behbahani, Alexander von Ruecker, Stefan C Müller, Jörg Ellinger

**Affiliations:** 1 Klinik und Poliklinik für Urologie und Kinderurologie, Universitätsklinikum Bonn, Bonn, Germany; 2 Institut für Pathologie, Universitätsklinikum Bonn, Bonn, Germany; 3 Hämatoonkologie, MVZ Labormedizin, Cologne, Germany; 4 Klinik und Poliklinik für Urologie und Kinderurologie, Universitätsklinikum Bonn, Sigmund-Freud-Strasse 25, 53105, Bonn, Germany

**Keywords:** Prostaglandin receptor, PTGER1, EP1, Ureter, Cyclooxygenase

## Abstract

**Background:**

Prostaglandins play an important role in ureteral obstruction, but the detailed expression profiles of the prostaglandin receptors (PTGER1, PTGER2, PTGER3, PTGER4, PTGFR) remain unknown in the different parts of the human ureter.

**Methods:**

The expression pattern of PTGER1, PTGER2, PTGER3, PTGER4 and PTGFR was determined in human distal, mid and proximal ureter and renal pelvis samples using immunohistochemistry (protein levels) and quantitative real-time PCR (mRNA).

**Results:**

PTGER1 was highly expressed in most samples irrespective of the ureteral localization; however, urothelial cells had higher levels of PTGER1 than smooth muscle cells. PTGFR was also moderately to strongly expressed in urothelial and smooth muscle cells. In comparison, PTGER2-4 expression was mostly unexpressed or weakly expressed in urothelial and smooth cells in all regions.

**Conclusions:**

Our data indicate high levels of PTGER1 in ureters.

## Background

Inhibition of cyclooxygenase (COX) activity and the concurrent reduction of prostaglandin synthesis via non-steroidal anti-inflammatory drugs (NSAIDs) are reported to reduce pain, pressure and ureteral contractility in patients experiencing ureteric colic or obstruction [1]. The synthesis of prostaglandins in patients with obstructed ureters contributes to alterations in renal hemodynamic function during ureteral obstruction, thus recent studies indicate NSAIDs to be beneficial in these patients. Especially COX2 mRNA has been shown to be upregulated in obstructed ureters and represents a valuable pharmaceutical target in patients with urolithiasis [2]. Potential toxic side effects of COX inhibitors include a decrease in renal perfusion, inhibition of platelet aggregation and gastric ulcerations. Beside COX-inhibitors, prostanoids (PG) represent an estimable physiological target in obstructed ureters: Granting that PGs constitute an heterogeneous group, PGF2a (prostaglandin F2a) and PGD2 (prostaglandin D2) cause ureteral contractility, PGE2 (prostaglandin E2) acts condition-dependent via four receptor subtypes (PTGER1-4) while PTGER1 (prostaglandin E receptor 1; alias EP1) and PTGER3 (prostaglandin E receptor 3; EP3) induce smooth-muscle contractility and PTGER2 (prostaglandin E receptor 2; EP2) and PTGER4 (prostaglandin E receptor 4; EP4) contribute to smooth muscle relaxation. PGE2-receptors represent G-protein coupled receptors which act via different signal transduction pathways: cAMP stimulation via Gq (PTGER2 and PTGER4), cAMP inhibition via Gi (PTGER3) or activation via phosphatidylinositol hydrolysis (PTGER1) [3]. The existence of PGE2-receptor subtypes has been verified in numerous animal models [1], yet the distribution of PGE2 receptors in the human urinary tract is not sufficiently examined. The impact of PTGER2 and PTGER3 is subject to further present studies, investigating the suppression of cytokine production [4] and the modulation of bone-related diseases [5]. Our study investigates the expression profiles of PTGER1-4 and PTGFR (prostaglandin F2 alpha receptor) in urothelial and smooth muscle cells in the renal pelvis, proximal, mid and distal human ureter. High expression profiles of PTGER1 and PTGER3 in obstructed ureters could contribute to new treatment approaches, complementing the therapy of urolithiasis as an alternative to the widespread use of COX inhibitors to provide superior efficacy while minimizing potential side effects of current therapy strategies.

## Methods

### Tissue samples

Tissue samples were obtained from 17 patients with malignant kidney tumors (renal cell carcinoma and urothelial cell carcinoma of the renal pelvis) and 6 patients with non-malignant disease undergoing nephroureterectomy for hydronephrotic kidney (chronic pyelonephritis, n=1; vesicoureteral reflux, n=2; obstructive megaureter, n=1; ureteropelvic junction obstruction, n=1; unknown etiology, n=1). Separate samples from the proximal ureter, mid ureter, distal ureter and renal pelvis of the above mentioned patients were embedded in paraffin after formalin-fixation (FFPE). Ureter samples from 3 patients were archived as fresh-frozen (FF) tissue and stored at −80°C. All tissue samples were collected within the framework of the Biobank at the Universitätsklinikum Bonn. All patients gave written informed consent prior to the collection of tissues. This study has been approved by the ethical committee of the University of Bonn, Germany (approval number 036/08).

### Immunohistochemistry

FFPE tissues were deparaffinized with xylol/ethanol and rehydrated; FF sections were warmed up at room temperature and rehydrated. The slides were then placed in citrate buffer (pH 5.0-6.0) for optimal labeling and cooked in a microwave for 20 minutes at 600 W. After cooling for 30 minutes, the slides were rehydrated, incubated in hydrogen peroxide for 10 minutes to block the peroxidase and washed with Tris-buffered saline with Tween (0.01% Tween 20, pH 7.6). The antibodies (PTGER1: Cayman, #101740, dilution 1:100; PTGER2: Sigma-Aldrich, #P7747, 1:500; PTGER3: Sigma-Aldrich, #HPA010689, 1:80; PTGER4: Sigma-Aldrich, #HPA012756, 1:100; PTGFR: Sigma-Aldrich, #NLS3890, 1:200; IgG: Dako, # P8622) were solved in Antibody Diluent (Dako, # K6008) and incubated overnight at 4°C. The slides were then washed with Tris-buffered saline with Tween. Afterwards, the EnVision Kit (Dako, #K4003) was used for visualization. Counterstaining was performed with haematoxylin. Staining was evaluated by a single investigator (M.O.). Human benign uterus was used as negative and kidney as positive control. In preliminary experiments, we have also investigated the expression pattern of target proteins in bladder, renal, ovary, colon and liver tissue (data not shown). The staining patterns were analogous to literature descriptions.

### Quantitative Real-Time PCR

RNA was isolated from FFPE and FF tissue of the renal pelvis, proximal ureter, mid ureter and distal ureter using the RecoverAll Total Nucleic Acid Isolation Kit for FFPE (Ambion, Foster City, CA, USA) according the manufacturers recommendations. We did not perform laser microdissection for separate analysis of urotheliale and muscle cells because of the low amount of tissue. Reverse transcription was performed using the SuperScript III Reverse Transcriptase Kit (Invitrogen, Carlsbad, CA, USA) and quantitative real-time PCR was performed using the SYBR GreenER Kit (Invitrogen); primer sequences are listed in Table 1. Each plate included a positive (kidney tissue) and negative control (uterus) and a water blank. PCR experiments were done with an ABI Prism 7900HT (Applied Biosystems, Foster City, CA, USA).

**Table 1:** Primer sequences

**Gene**	**Forward primer**	**Reverse primer**
**GAPDH**	CCCCGGTTTCTATAAATTGAGC	CACCTTCCCCATGGTGTCT
**ACTB**	CCAACCGCGAGAAGATGA	CCAGAGGCGTACAGGGATAG
**PTGER1**	ATGGTGGTGTCGTGCATCT	CGCTGCAGGGAGGTAGAG
**PTGER2**	CCACCTCATTCTCCTGGCTA	AGGTCCCATTTTTCCTTTCG
**PTGER3**	ATCATGTGCGTGCTGTCG	TGCAGTGCTCAACTGATGTCT
**PTGER4**	CTCCCTGGTGGTGCTCAT	GGCTGATATAACTGGTTGACGA
**PTGFR**	GGGATCTACAGCCAGACCAG	GTGTCCTATGTCCTCCAAACG

### Statistical analysis

All statistical tests were performed using IBM SPSS Statistics v20. The chi-square test was used to compare protein expression levels in urothelial and smooth muscle cells, and different parts of the human ureter respectively. The Friedman-test for paired-samples was applied to compare protein expression levels in different ureter samples. Statistical significance was concluded at p < 0.05.

## Results

### Immunohistochemistry

Initially, we compared immunohistochemical staining of FF and FFPE tissue. The evaluation of FF tissue staining results was limited due to the fact that urothelial cells detached from muscle tissue. Thus, we restricted the analysis below to FFPE tissue. It should be noted that staining results of muscle cells were similar in FF and FFPE tissues.

**Figure 1:**
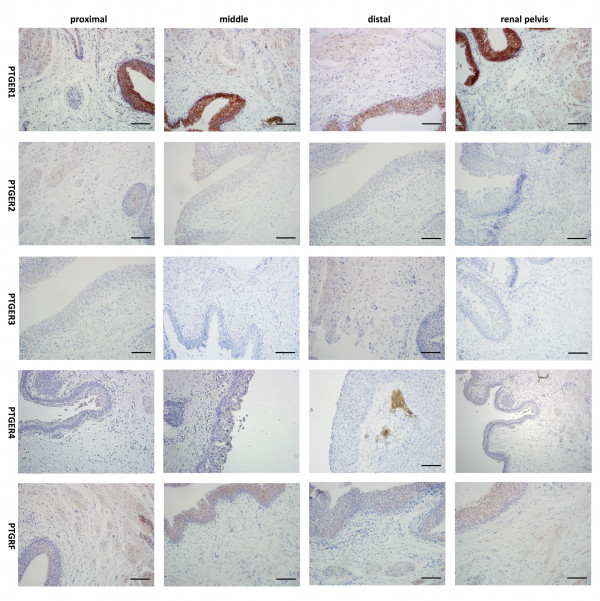
Representative staining of PTGER1-4 and PTGFR in the human ureter and renal pelvis.

**Figure 2:**
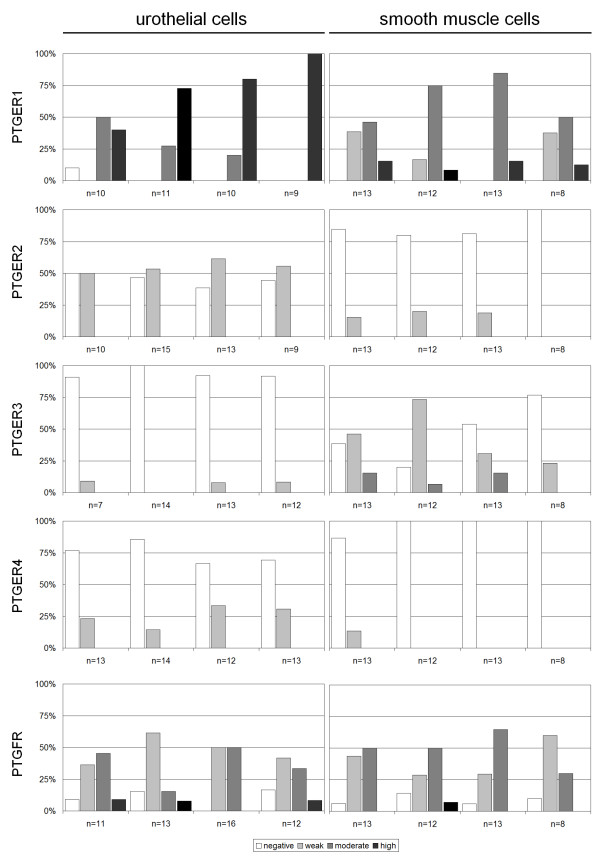
Bar diagrams demonstrate expression levels of PTGER1-4 and PTGFR in urothelial and smooth muscle cells of the human ureter.

We observed cytoplasmic expression of PTGER1-4 and PTGFR (see Figure 1). The target proteins were detected in all cells at homogenous intensity within a given sample, and therefore protein expression was scored negative, low, moderate or high in a sample. We did not notice expression differences in obstructed and non-obstructed ureter tissues (p > 0.28, Mann–Whitney-U-test). The expression of each target protein was markedly different in the ureters (see Figure 2), and also the expression level of the proteins was different in the various parts of the ureter and renal pelvis (Friedman-test: p < 0.001; see Additional file 1: Table S1 for expression profiles in the different samples):

- *PTGER1* was expressed in most samples, but expression was increased in urothelial compared to smooth muscle cells. Most samples demonstrated high PTGER1 expression levels in urothelial cells, whereas muscle cells were expressing PTGER1 at moderate levels. Further analysis of PTGER1 expression profiles depicted comparable expression profiles in the renal pelvis, as well as the lower, mid and proximal ureter.

- *PTGER2* expression was less prevalent: Urothelial cells demonstrated limited staining, irrespective of the localization in the examined ureter. In comparison, PTGER2 expression profiles showed weak staining of muscle cells in 15-20% of the ureter samples. PTGER2 expression was shown to be undetected in muscle cells located in the renal pelvis.

- *PTGER3* was rarely detected in urothelial cells (10%) of the renal pelvis and the distal and proximal ureter. In comparison, PTGER3 was more prevalent in the smooth muscle cells, with different expression profiles, depending on the ureter localization: Expression profiles were lower in the upper parts of the examined ureters. In total, 62% of the distal ureter, 80% of the mid ureter, 46% of the proximal ureter and 23% of the renal pelvis samples expressed PTGER3 at low or moderate levels.

- PTGER4 expression was low, and detected more frequently in urothelial cells than in muscle cells. Only 13% of distal ureter muscle cells were expressing PTGER4 at low levels. PTGER4 expression was also low in urothelial cells, but detected in the distal ureter (23%), mid ureter (14%), proximal ureter (33%) and renal pelvis (31%).

- *PTGFR* expression was detected in most of the ureter samples: Less than 15% of the samples had undetectable PTGFR levels. The expression of PTGFR was similar in urothelial and smooth muscle cells.

### Real-Time PCR

We next investigated the expression profiles of PTGER1-4 and PTGFR mRNA in ureter tissue. We first tried to examine mRNA in FFPE materials. Most probably due to the low amount of available material and the consequences of formalin-fixation, the amount and quality of RNA was not sufficient for PCR experiments: We did neither detect any target gene nor the reference genes ACTB and GAPDH in the ureter tissue. We therefore used FF tissue for PCR. Again, the low amount of each tissue impeded the mRNA quantification: the expression levels of the target genes were only marginal above the quantification limit (Cq values 30 to 32); the stability values for ACTB and GAPDH were 0,538 (as determined using the DataAssist software). All three samples had detectable amounts of PTGER1-4 and PTGFR mRNA. But, mRNA and protein levels (detected by immunohistochemistry in adjacent FF tissue) were not correlated (all p > 0.3). Small amount of mRNA were even detected in samples without traceable corresponding protein. See Table 2.

**Table 2:** Comparison of prostaglandin receptors’ mRNA and protein levels in different ureteral locations

	**PTGER1**		**PTGER2**		**PTGER3**		**PTGER4**		**PTGFR**	
	**PCR**	**IHC**	**PCR**	**IHC**	**PCR**	**IHC**	**PCR**	**IHC**	**PCR**	**IHC**
A-distal	1.00	+++	1.00	++	1.00	-	1.00	+	1.00	+++
A-middle	1.44	++	0.31	+	0.37	+++	1.27	-	0.34	++
A-proximal	3.15	+++	0.62	+	0.42	-	1.09	+	0.64	++
A-renal pelvis	2.73	++	0.55	-	0.36		1.63		0.55	
B-distal	0.61	++	0.14	++	0.69	++	0.67	-	0.31	+
B-middle	0.41	++	0.22	++	0.95	++	0.87	-	0.31	++
B-proximal	0.21	++	0.20	+	1.04	++	0.83	-	0.30	+++
B-renal pelvis	1.09	++	0.30	+	0.76	+	1.00		0.33	
C-distal	1.28	+++	0.46	++	0.73	++	0.81	-	0.59	+++
C-proximal	1.11	++	0.36	++	1.23	++	0.84	-	0.64	++
C-renal pelvis	3.51	+	0.29	+	2.14	+	1.80		0.49	

Note: Sample A: pyelonephritis, non-obstructed; Sample B: hydronephrotic kidney, non-obstructed; Sample C: renal cella carcinoma, non-obstructed

Abbreviations: - = −; + = +; ++ = ++; +++ = +++; = tissue not available.

## Discussion

The analysis of prostaglandin receptor expression profiles in obstructed human ureters plays an important role in the attempt to implement new therapy strategies with better efficiency and reduced side effects in comparison to current standards. Previous studies have already documented the role and significance of prostaglandin receptors in animal models [1], leaving the need to analyze distribution profiles in the human urinary tract. By now, non-steroidal anti-inflammatory drugs (NSAIDs) are widespread used against acute pain. Especially COX2 has been demonstrated to show an increased expression in obstructed ureters and represents a valuable target for selective COX2 inhibitors, such as parecoxib [1]. Selective and non-selective COX-inhibitors have many side effects such as gastrointestinal side effects and a promotion of bleeding [6]. NSAIDs are also under suspicion to cause renal dysfunction, such as salt and fluid retention and hypertension [7]. Even acute renal failure is a possible adverse reaction [8]. In the view of potential side effects, recent studies have demonstrated the effect of PGE2 on obstructed ureters: Lowry et al. showed that PGE2 increases contractility in obstructed ureters and relaxes non-obstructed ureters [9]. The demonstrated condition-dependence of PGE2 receptors might be contributed to its various subtypes [10]: While Regan et al. [11] report that PTGER2 and PTGER4 subtypes relax smooth-muscle contractility, Neves et al. [12] showed a contractile effect, mediated by PTGER1 and PTGER3. Our study demonstrates high expression profiles of PTGER1 in urothelial cells of the renal pelvis (100%) as well as the proximal ureter (80%) decreasing rates to the distal part of the ureter. Accordingly, smooth muscle cells showed moderate to high expression profiles of PTGER1, especially in the proximal part of ureters (85%). So far, PTGER1 expression profiles have sufficiently been examined for bladder urothelial cells [13]. Ikeda et al. state that PTGER1 receptors in the urothelium of the bladder may facilitate the inflammation-induced micturition reflex via primary nerve activity [13]. On the other hand Johansson et al. were recently able to demonstrate the significant contribution of PTGER1 receptors to inflammation induced pain [14]. Interestingly, PTGER1 receptors play an important role in resolving inflammation through down-regulation of COX2 [15]. This aspect, along with strong expression profiles of PTGER1 in our study, underlines the need to introduce a selective PTGER1 antagonist complementary to the use of COX2 inhibitors.

PTGER1 receptor antagonists have been shown to have an excellent profile in inflammatory models [16]. So far, the activation of PTGER1 has been shown to specifically activate the action of aldosterone on epithelial sodium channels expression in the renal medulla [17]. Concurrently, Ankem et al. [10] indicate that the PTGER3 subtype of the PGE2 receptor is involved in hypercontractility during ureteral obstruction. However, the authors conclude that PTGER3 is not the sole factor contributing to the condition dependency of prostaglandin receptors. Our study shows consistent results with weak to moderate expression profiles of PTGER3 in smooth muscle cells of the proximal, mid and distal ureter, as well as negative expression in urothelial cells. PTGER3 receptors may function via cAMP response elements (CRE) [18] and have been shown to be useful targets for anti-inflammatory therapy in cutaneous inflammation [19]. In contrast, PTGER2 and PTGER4 subtypes showed negative to weak expression profiles in urothelial as well as smooth muscle cells.

Some limitations of our study need to be discussed: upper urinary tract tissue samples were obtained from patients with different pathologies (cancer, chronic pyelonephritis, vesicoureteral reflux, obstructive megaureter, ureteropelvic junction obstruction) and obstructive and non-obstructive ureters. Associated chronic inflammatory states - although excessive infiltration of tissues with leukocytes was not seen - altering prostaglandin receptor expression patterns may influence the results. Furthermore, PCR experiments were hindered by RNA degradation, and data interpretation has to be done with caution. It has to be mentioned that the collection of large numbers of urinary tract samples with an homogenous clinic is difficult; a larger cohort might demonstrate differences of prostaglandin receptor expression in obstructive and non-obstructive ureters.

## Conclusions

High expression levels of PTGER1 in obstructed ureters support rational drug development, as e.g. EPS antagonists, to complement complement the treatment of patients with urolithiasis as an alternative to the widespread and unselective use of COX2 inhibitors to provide superior efficiency while minimizing potential side effects.

## Competing interests

The study was supported by an grant from Astellas Pharmaceuticals (Leiderdorp, Netherlands).

## Authors’ contributions

MO carried out the experiments. The manuscript was written by MO, CB, TEB and JE. MO and JE performed the statistical analyses. SCM, AR and JE participated in the study design. All authors read and approved the final manuscript.

## Pre-publication history

The pre-publication history for this paper can be accessed here:

http://www.biomedcentral.com/1471-2490/12/35/prepub

## Supplementary Material

Additional file 1: Table S1 Overview of prostaglandin receptor expression in individual ureter samples. Click here for file

## References

[B1] Nørregaard R Jensen BL Topcu SO Nielsen SS Walter S Djurhuus JC Frøkiaer J Cyclooxygenase type 2 is increased in obstructed rat and human ureter and contributes to pelvic pressure increase after obstruction Kidney Int 2006 70 5 872 881 10.1038/sj.ki.5001616 16820795

[B2] Chaignat V Danuser H Stoffel MH Z'brun S Studer UE Mevissen M Effects of a non-selective COX inhibitor and selective COX-2 inhibitors on contractility of human and porcine ureters in vitro and in vivo Br J Pharmacol 2008 154 6 1297 1307 10.1038/bjp.2008.193 18500363 PMC2483393

[B3] Breyer MD Jacobson HR Breyer RM Functional and molecular aspects of renal prostaglandin receptors J Am Soc Nephrol 1996 7 1 8 17 8808104 10.1681/ASN.V718

[B4] Ueta M Matsuoka T Yokoi N Kinoshita S Prostaglandin E2 suppresses polyinosine-polycytidylic acid (polyI:C)-stimulated cytokine production via prostaglandin E2 receptor (EP) 2 and 3 in human conjunctival epithelial cells Br J Ophthalmol 2011 95 6 859 863 10.1136/bjo.2010.199679 21349943

[B5] Noh AL Yang M Lee JM Park H Lee DS Yim M Phosphodiesterase 3 and 4 negatively regulate receptor activator of nuclear factor-kappaB ligand-mediated osteoclast formation by prostaglandin E2 Biol Pharm Bull 2009 32 11 1844 1848 10.1248/bpb.32.1844 19881295

[B6] Labianca R Sarzi-Puttini P Zuccaro SM Cherubino P Vellucci R Fornasari D Adverse effects associated with non-opioid and opioid treatment in patients with chronic pain Clin Drug Investig 2012 32 Suppl 1 53 63 10.2165/11630080-000000000-00000 22356224

[B7] Bao H Ge Y Zhuang S Dworkin LD Liu Z Gong R Inhibition of glycogen synthase kinase-3beta prevents NSAID-induced acute kidney injury Kidney Int 2012 81 7 662 673 10.1038/ki.2011.443 22258319 PMC3305839

[B8] Musu M Finco G Antonucci R Polati E Sanna D Evangelista M Ribuffo D Schweiger V Fanos V Acute nephrotoxicity of NSAID from the foetus to the adult Eur Rev Med Pharmacol Sci 2011 15 12 1461 1472 22288307

[B9] Lowry PS Jerde TJ Bjorling DE Maskel JL Nakada SY Obstruction alters the effect of prostaglandin E2 on ureteral contractility J Endourol 2005 19 2 183 187 10.1089/end.2005.19.183 15798415

[B10] Ankem MK Jerde TJ Wilkinson ER Nakada SY Third prize: Prostaglandin E(2)-3 receptor is involved in ureteral contractility in obstruction J Endourol 2005 19 9 1088 1091 10.1089/end.2005.19.1088 16283845

[B11] Regan JW Bailey TJ Pepperl DJ Pierce KL Bogardus AM Donello JE Fairbairn CE Kedzie KM Woodward DF Gil DW Cloning of a novel human prostaglandin receptor with characteristics of the pharmacologically defined EP2 subtype Mol Pharmacol 1994 46 213 220 8078484

[B12] Funk CD Furci L Fitzgerald GA Grygorczyk R Rochette C Bayne MA Abramovitz M Adam M Metters KM Cloning and expression of a cDNA for the human prostaglandin E receptor EP1 subtype J Biol Chem 1993 268 26767 26772 8253813

[B13] Wang X Momota Y Yanase H Narumiya S Maruyama T Kawatani M Urothelium EP1 receptor facilitates the micturition reflex in mice Biomed Res 2008 29 2 105 111 10.2220/biomedres.29.105 18480552

[B14] Johansson T Narumiya S Zeilhofer HU Contribution of peripheral versus central EP1 prostaglandin receptors to inflammatory pain Neurosci Lett 2011 495 2 98 101 10.1016/j.neulet.2011.03.046 21440042

[B15] Haddad A Flint-Ashtamker G Minzel W Sood R Rimon G Barki-Harrington L Prostaglandin EP1 Receptor Down-regulates Expression of Cyclooxygenase-2 by Facilitating Its Proteasomal Degradation J Biol Chem 2012 287 21 17214 17223 10.1074/jbc.M111.304220 22474323 PMC3366802

[B16] Giblin GM Bit RA Brown SH Chaignot HM Chowdhury A Chessell IP Clayton NM Coleman T Hall A Hammond B Hurst DN Michel AD Naylor A Novelli DR Coccitti T Spalding D Tang SP Wilson AW Wilson R The discovery of 6-[2-(5-chloro-2-{[(2,4-difluorophenyl)methyl]oxy}phenyl)-1-cyclopenten-1-yl]-2-pyridinecarboxylic acid, GW848687X, a potent and selective prostaglandin EP1 receptor antagonist for the treatment of inflammatory pain Bioorg Med Chem Lett 2007 17 2 385 389 10.1016/j.bmcl.2006.10.041 17084082

[B17] González AA Céspedes C Villanueva S Michea L Vio CP E Prostanoid-1 receptor regulates renal medullary alphaENaC in rats infused with angiotensin II Biochem Biophys Res Commun 2009 389 2 372 377 10.1016/j.bbrc.2009.08.157 19732740

[B18] Audoly LP Ma L Feoktistov I de Foe SK Breyer MD Breyer RM Prostaglandin E-prostanoid-3 receptor activation of cyclic AMP response element-mediated gene transcription J Pharmacol Exp Ther 1999 289 1 140 148 10086997

[B19] Goulet JL Pace AJ Key ML Byrum RS Nguyen M Tilley SL Morham SG Langenbach R Stock JL McNeish JD Smithies O Coffman TM Koller BH E-prostanoid-3 receptors mediate the proinflammatory actions of prostaglandin E2 in acute cutaneous inflammation J Immunol 2004 173 2 1321 1326 15240726 10.4049/jimmunol.173.2.1321

